# 双克隆型多发性骨髓瘤患者的临床生物学特征及预后

**DOI:** 10.3760/cma.j.issn.0253-2727.2021.08.011

**Published:** 2021-08

**Authors:** 薇 程, 玉龙 李, 洲风 黄, 曌博 李, 晓燕 董, 保军 商, 琳 张, 杰 时, 尊民 朱

**Affiliations:** 河南省人民医院血液病研究所，郑州大学人民医院血液科，河南省干细胞调控及分化重点实验室 450003 Department of Hematology, Zhengzhou University People's Hospital, Institute of Hematology, Henan Province People's Hospital, Henan Key Laboratory of Stem Cell Regulation and Differentiation, Zhengzhou 450003, China

多发性骨髓瘤（MM）是一种浆细胞恶性增殖性疾病，约占血液系统恶性肿瘤的10％[1]，根据免疫固定电泳分析，MM可分为IgG、IgA、IgM、IgD、IgE、轻链型、双克隆型以及不分泌型[2]。分泌双克隆免疫球蛋白的MM是一种少见的浆细胞病，发病率较低，国内尚缺乏大宗报道。为进一步了解双克隆型MM的临床生物学特征及预后，我们回顾性分析了20例双克隆型MM患者的临床特点、生物学特征及预后，报道如下。

## 病例与方法

1. 病例：回顾性分析了2015年1月至2020年6月河南省人民医院收治的20例双克隆型MM患者的临床资料，诊断均符合2014年国际骨髓瘤工作组（IMWG）的诊断标准。所有患者均进行血常规、生化常规、免疫固定电泳、骨髓细胞形态学、流式细胞术、FISH检测，对其临床生物学特征及预后等进行分析。

2. 免疫固定电泳检测：采集患者外周血血清，应用美国Helena公司全自动快速电泳分析系统及配套原装试剂进行免疫固定电泳检测，并根据电泳结果进行免疫分型。

3. 骨髓细胞学检查：所有患者行骨髓穿刺后进行骨髓细胞形态学分析，行瑞特-吉姆萨染色，按法-美-英（FAB）分型方法在镜下进行分类，计数250个有核细胞，并计算骨髓瘤细胞所占百分比。

4. 免疫表型检测：所有患者均抽取肝素抗凝骨髓液2 ml，按照多色免疫荧光直接标记法操作。荧光标记鼠抗人单克隆抗体（CD45-KO、CD38-APCCY7、anti-Kappa-FITC、CD138-APC、CD56-PE CY7、CD22-PE、CD117-PerCP5.5、anti-Lambda-PE、CD20-PB、CD19-PE CY7、CD19-ECD、HLA-DR-FITC、CD34-PerCP5.5、CD38-APC）（美国Beckman Coulter公司产品），细胞标记后上流式细胞仪（Navios，美国Beckman Coulter公司产品）检测，获取500 000个细胞。通过CD45/SSC、CD38/SSC和CD38/CD45联合设门圈定待测细胞群，并分析该群细胞抗原表达情况。抗原表达≥20％定义为抗原阳性。

5. FISH检测：分别抽取初诊患者肝素抗凝的骨髓标本5～10 ml，采用密度梯度离心法分离骨髓单个核细胞，磁珠分离CD138阳性细胞。观察200个分裂间期细胞荧光杂交信号，包括1q+、17p−、13q−、t（11;14）、t（4;14）、t14;16），进行图像分析。按照欧洲骨髓瘤工作组设定的阳性阈值标准[3]：13q−、17p−和1q+的阳性阈值为20％，融合基因的阳性阈值为10％。基于2018年更新的危险分层标准（mSMART3.0），双/三打击定义为具有高危遗传学异常中的任意两种或三种。

6. 治疗及疗效评估：年龄≤65岁且适合自体造血干细胞移植者，予PAD（硼替佐米+阿霉素+地塞米松）或PCD（硼替佐米+环磷酰胺+地塞米松）方案化疗。年龄≥65岁或不适合自体造血干细胞移植者，予PCD或BD（硼替佐米+地塞米松）方案化疗，部分高龄患者根据其耐受程度适当减量。所有患者均未接受造血干细胞移植。4个疗程结束后参照中国多发性骨髓瘤诊治指南（2020年修订版）[2]进行疗效评估。疗效分为完全缓解（CR）、非常好的部分缓解（VGPR）、部分缓解（PR）、疾病稳定（SD）、疾病进展（PD）。客观缓解率（ORR）为CR率、VGPR率与PR率之和。

7. 随访：随访日期截至2020年12月31日，中位随访26（3～62）个月。1例患者失访，失访病例按末次明确存活时间处理，末次随访仍存活者按截尾数据处理。总生存（OS）时间指患者从确诊至死亡或末次随访的时间。无进展生存（PFS）时间指患者自确诊至疾病进展或末次随访的时间。早期死亡指患者确诊后6个月内发生的死亡。

## 结果

1. 一般临床特征：20例患者均明确诊断为双克隆型MM，占我院同期692例MM患者的2.89％，其中男13例，女7例，男女比例为1.86∶1。年龄构成方面，≥60岁患者10例（50％），60岁以下患者10例（50％）。临床分期R-ISSⅠ期患者1例（5％），Ⅱ期患者16例（80％），Ⅲ期患者3例（15％）。IgG λ-IgA λ型11例（55％），IgG κ-IgA κ型5例（25％），IgG κ-IgG λ型3例（15％），IgG κ-IgA λ型1例（5％）。骨髓浆细胞比例≥30％患者8例（40％）、<30％患者12例（60％）。

2. 血液学检查结果：14例（70％）患者HGB<100 g/L，且以轻度贫血多见。生化常规结果显示肾功能不全患者6例（30％）。20例患者β_2_-微球蛋白水平均增高，其中≥5.5 mg/L者7例（35％）。血清钙水平正常者多见，仅3例（15％）患者为高钙血症。

3. 免疫表型：所有患者均进行流式细胞术检查，所有患者均表现出一定程度的免疫表型异常，异常浆细胞均表达CD38、CD138，但表达强度在流式细胞术散点图上表现为较正常浆细胞减弱。频率最高的异常是CD19表达缺失（100％）及CD56异常表达（55％）。而CD117及HLA-DR表达例数分别为6例和4例，分别占35％和20％。CD45表达呈阴性或弱阳性患者16例，表达阳性患者4例（20％）。cκ阳性9例，cλ阳性11例，胞质轻链阳性患者比例为cκ∶cλ＝0.82∶1。CD56、CD117双阴性9例（45％）。而CD34、CD20均阴性。

4. 细胞遗传学特征：患者FISH检查结果示amp1q21 11例，13q− 9例，t（4;14）4例，t（11;14）3例，17p− 2例，t（14;16）2例，双打击及三打击患者共5例。

5. 疗效及生存情况：19例患者完成4个疗程以上的化疗，4个疗程结束后患者的疗效评估显示，双克隆型MM患者ORR为57.89％，其中CR 1例、VGPR 4例、PR 6例、SD 6例、PD 2例。双克隆型MM患者中位OS时间为28（95％*CI* 3～53）个月，中位PFS时间为12（95％*CI* 2～39）个月，随访截止时双克隆型MM患者死亡5例，死亡率为25％，6个月以内的早期死亡患者3例，早期死亡率为15％。

## 讨论

MM是一种浆细胞恶性增殖性疾病。通常，MM患者血清和（或）尿液中的单克隆免疫球蛋白是由一种重链和一种轻链构成，但有2％～6％患者的血清和（或）尿液中会出现两种及以上类型的单克隆免疫球蛋白，在免疫固定电泳上出现两个不同的异常免疫球蛋白条带，即为双克隆型MM[4]。鉴于其罕见性，目前关于双克隆型MM的大宗报道少见，对此类MM患者的临床生物学特征、治疗反应、预后等尚缺乏深刻的认识[5]。我中心2015-2020年新诊断的692例MM患者中有20例双克隆型MM，发生率约为2.89％。

本研究患者均以双重链和双轻链形式呈现，重链以IgG和IgA型为主，主要类型为IgA λ-IgG λ型，与既往相关报道中M蛋白的组合形式及常见类型有所不同[5]–[8]。既往双克隆型MM的报道及本研究显示，双克隆型MM以何种组合多见尚无一定规律，所见报道各有差异。有文献认为双克隆型MM的区别主要取决于轻链类型，与两种重链亚型无关，同时表达κ和λ轻链的病例发生率极高[9]。但国内报道并不多见，我们也仅见到4例M成分轻链不同的病例。

多色流式细胞术、细胞遗传学检查在MM诊断、预后判断中的重要价值已被临床广泛认识。本研究流式细胞术结果显示双克隆型患者异常浆细胞免疫表型与单克隆型MM基本类似，CD45^−^ CD19^−^ CD56^+^伴胞质轻链限制性表达是最常见的异常，未见同时表达κ和λ的肿瘤细胞或两种轻链不同的肿瘤细胞存在。此结果和免疫固定电泳在轻链分布方面具有一定差异，可能与小克隆被优势克隆掩盖导致流式细胞术检测灵敏度偏低有关。我们早期研究发现CD45阳性及CD56/CD117双阴性均是MM患者的预后不良因素[10]–[11]。本研究CD45阳性比例和我们早期研究基本一致，但CD56/CD117双阴性所占比例则明显高于既往结果[10]–[11]。双克隆型MM的遗传学异常也很常见，遗传学异常所占比例基本与单克隆型MM一致[12]，但明显高于国外报道的17％[5]，可能与地域、民族及MM类型有关[5],[12]。另有研究提示携带多种高危遗传学异常患者的预后明显劣于携带一种高危遗传学异常的患者，多种不良预后因素组合导致患者的预后和生存更差[13]–[14]。在我们的研究中，双克隆型MM患者中双/三打击的发生率为25％，不良预后因素可能导致此类患者预后较差。但关于双克隆型MM免疫表型及细胞遗传学方面的文献报道较少，且我们的病例数有限，所以相关结果有待大样本研究进一步证实。

本研究双克隆型MM患者4个疗程后ORR为57.80％，低于Jurczyszyn等[5]的研究结果，但Jurczyszyn等[5]的研究是基于4年的治疗反应，一线治疗方案多样且包括部分造血干细胞移植的患者，因此与我们的短期疗效分析结果有一定差异。我们发现一部分双克隆型MM患者会发生早期死亡，约占10.53％。双克隆型MM早期疗效欠佳及早期死亡是否与其不良预后表型及双/三打击有关有待更大样本的进一步研究证实。虽然有研究认为第二个克隆的存在或出现与更好的治疗反应、缓解率和较慢的进展有关[15]–[16]，但另一些研究并不支持此结果[5],[9],[17]。Jurczyszyn等[5]发现无论以何种方案治疗，双克隆型MM在治疗反应及生存分析方面与单克隆型MM无显著差异，双克隆本身并不是MM患者预后不良的独立危险因素。与既往报道相比，本研究患者的整体死亡率、PFS率及OS率均偏低，由于样本量的限制，结果尚待进一步证实[5]。

综上所述，双克隆型MM是一种少见的MM类型，中位发病年龄约60岁，临床特征不特异，诊断需依赖免疫固定电泳等相关实验室检查。综合国内外报道，该类型患者的临床预后与单克隆型MM相比尚未发现明显差异，但本研究发现此类型患者早期疗效不佳，并有一定比例的患者发生早期死亡，可能与部分患者具有不好的免疫表型及高危遗传学异常有关。因此，我们认为老年MM患者应警惕双克隆型MM的发生，进行流式细胞术免疫分型及FISH等全面的实验室检查，尽量做到早确诊、早治疗、早评估，以提高此类患者的疗效。
